# Development and application of a 2-step methodology to select a reference society providing Dietary Reference Values for national implementation

**DOI:** 10.1017/S1368980023002902

**Published:** 2024-01-02

**Authors:** Corinne Jotterand Chaparro, Clémence Moullet, Valeria A Bertoni Maluf, Nicolas Parel, Lyvonne N Tume, Angeline Chatelan, Clara Benzi Schmid, Raphaël Reinert, Sophie Bucher Della Torre

**Affiliations:** 1 Department of Nutrition and Dietetics, Geneva School of Health Sciences, HES-SO University of Applied Sciences and Arts Western Switzerland, Rue des Caroubiers 25, 1227 Carouge, Geneva, Switzerland; 2 Faculty of Health, Social Care & Medicine, Edge Hill University, Ormskirk, UK; 3 Swiss Federal Food Safety and Veterinary Office, Bern, Switzerland

**Keywords:** Dietary reference values, Development of methodology, National implementation, Macronutrients, Micronutrients

## Abstract

**Objective:**

To describe and discuss a 2-step methodology developed to select a reference society that provides Dietary Reference Values (DRV) for national implementation and to illustrate its application in Switzerland with one macronutrient and one micronutrient.

**Design:**

During Step 1, we searched and compared DRV and methodologies used to define DRV from eight European societies for seven selected nutrients. We repeated this procedure during Step 2 for DRV from two preselected societies for forty-four nutrients.

**Setting:**

The 2-step methodology applied here for Switzerland may be used in other countries.

**Participants:**

The research team commissioned six external experts from three linguistic regions of Switzerland, who provided their opinions through two online surveys, individual interviews and a focus group.

**Results:**

After Step 1, we excluded five societies because of old publication dates, irrelevant publication languages for Switzerland, difficulty in accessing documents, or because their DRV were mainly based on another society. After Step 2, the two societies were qualified based on the analysis of the values and methodologies used. The need for free and easily accessible scientific background information favoured the European Food Safety Authority (EFSA). We chose alternative societies for nine nutrients for the overall population or subgroups and for the elderly.

**Conclusions:**

To manage heterogeneous and complex data from several societies, adopting a 2-step methodology including fewer nutrients and more societies during Step 1, and fewer societies but all nutrients in Step 2, was very helpful. With some exceptions, we selected EFSA as the main society to provide DRV for Switzerland.

Dietary Reference Values (DRV) provide a scientific basis for developing nutritional and dietary recommendations. They guide professionals to assess the amount of a nutrient needed to maintain health in a healthy individual or a group of healthy people^(1)^. DRV are also essential for nutrition-related public health actions aiming to help consumers make positive choices for a balanced diet^(2)^. Among others, DRV serve to develop national or regional nutrition policies, nutritional education programmes, food labelling or food regulations^(3)^.

DRV is an umbrella term for a set of nutrient reference values that includes the Average Requirement, Population Reference Intake (PRI), Adequate Intake (AI), Reference Intake range for macronutrients, Tolerable Upper Intake Level and Lower Threshold Intake.

Many nutrition scientific institutions provide DRV, such as the WHO, the Institute of Medicine and the European Food Safety Authority (EFSA). There is no standard approach for establishing DRV. In Europe, most countries have DRV, either mainly developed on their own or based on existing DRV. For example, France and Belgium rely mainly on EFSA. Moreover, DRV are more or less regularly updated, depending on the country or nutrients.

In Switzerland, a small and multi-linguistic country (German, French or Italian) in the centre of Europe, the three main linguistic regions often used DRV published by different organisations. This includes the Società Italiana di Nutrizione Umana (SINU, Italy)^(4)^, the French Agency for Food, Environmental and Occupational Health & Safety (ANSES, France)^(5)^ and the D-A-CH reference values for nutrients jointly issued by the Nutrition Societies of Germany, Austria and Switzerland^(6)^. In addition, the Federal Commission for Nutrition (FCN) has developed specific DRV for the Swiss population for six nutrients: lipids (2013)^(7)^, protein (2011)^(8)^, carbohydrates (2009)^(9)^, vitamin D (2012)^(10)^, folate (2002)^(11)^ and iodine (2013)^(12)^. FCN also provided DRV for the majority of nutrients for the group of elderly (2018)^(13)^.

In this historical and cultural setting, the Swiss Federal Food Safety and Veterinary Office (FSVO) wished to have DRV based on solid and updated scientific data recognised throughout the country. Having updated, harmonised and nationwide DRV is important, especially in assessing the nutritional status of the population, monitoring nutritional intake and updating food-based dietary guidelines in a similar manner throughout the country. FSVO chose to use the existing DRV instead of developing new Swiss DRV for efficiency reasons and because the Swiss population shares the main physiologic characteristics than their European neighbours. They commissioned our research group to identify which reference societies may provide updated and appropriate DRV for the population living in Switzerland, including different ages, genders and pregnant and breastfeeding women. We developed a 2-step methodology to analyse the DRV of eight societies and provided FSVO with a scientific report containing our practical recommendations for Switzerland^(14)^. This paper aims to describe and discuss the 2-step methodology developed to select a reference society providing DRV for national implementation. In addition, we illustrate its application in Switzerland with one macro- (i.e. protein) and one micronutrient (i.e. iron), whose DRV may differ largely between age groups, genders and for pregnant and breastfeeding women.

## Methods

### Overview of the project

**Fig. 1:**
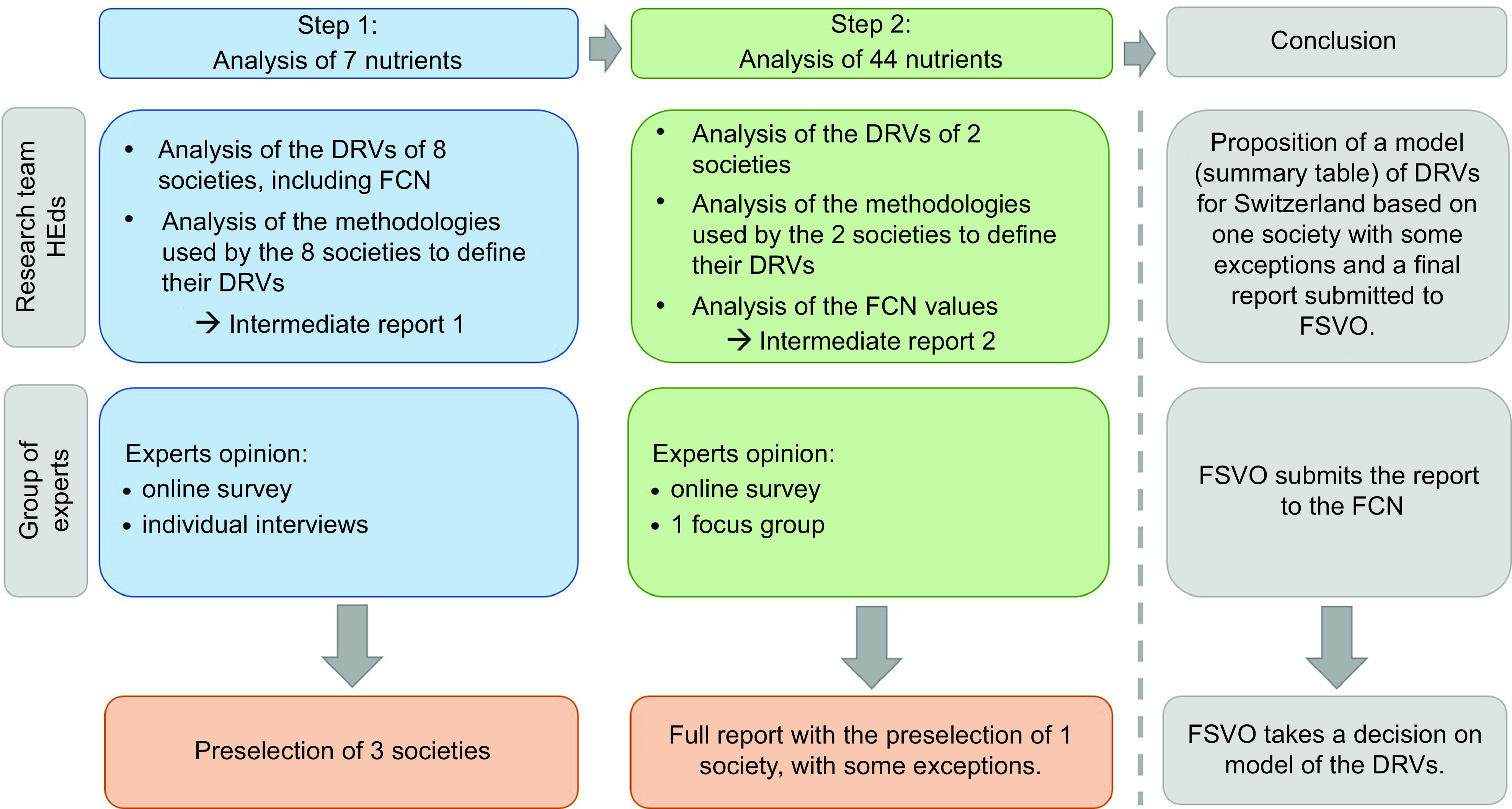
Flow chart showing the different steps of the project

The 2-step methodology and its main stages are shown in Fig. 1. During Step 1, from September 2020 to February 2021, we searched and compared the DRV from eight selected scientific societies and analysed the methodologies used to define the DRV. Due to the considerable amount of data, the analyses were conducted on seven selected nutrients: protein, carbohydrates, folate, vitamin D, calcium, iodine and iron. Three reasons motivated this choice: (i) their DRV may vary between subgroups, including gender, (ii) large variabilities in DRV were expected between societies, (iii) FCN provided DRV for the majority of these nutrients. The analyses were summarised in a report sent to six external experts from the three linguistic regions of Switzerland, with various backgrounds, scientific training, professional experience and knowledge of nutritional recommendations in the Swiss context. The experts participated in an online survey, followed by individual interviews to provide their opinions for the preselection of 2–4 societies for Step 2.

In Step 2, conducted from February to June 2021, we searched and compared the DRV of forty-four macronutrients and micronutrients from preselected societies. The findings were summarised in a second report submitted to the same group of experts. This was followed by a second online survey and a focus group. Finally, the research group made recommendations for FSVO in a final report^(14)^.

### Inclusion and exclusion criteria used to identify scientific societies

Before Step 1, the inclusion and exclusion criteria were used to identify eligible societies that could provide DRV for Switzerland. The inclusion criteria were societies that (i) updated their DRV in the last 10 years (since 2011); (ii) provided DRV (including Average Requirement, PRI, AI, Upper Intake Level and Lower Threshold Intake) for energy, all macronutrients and the majority of micronutrients; (iii) provided DRV for one or more European countries with a population close to the Swiss population in terms of socio-demographic characteristics and eating habits, as these factors do not influence the physiological needs, but may be taken into account to define the PRI; and (iv) were internationally recognised.

Societies that did not meet these criteria were excluded. Based on these criteria, we included eight societies in the project (detailed in Table 1): ANSES^(5)^, Superior Health Council of Belgium (SHC)^(15)^, D-A-CH^(6)^, EFSA^(1)^, FCN^(16)^, Nordic Nutrition Recommendations (NNR)^(17)^, Scientific Advisory Committee on Nutrition (SACN)^(18)^ and SINU^(4)^. FCN is an extra-parliamentary commission composed of experts in the field of nutrition who act in an advisory capacity. One of its tasks is to develop position papers that include recommendations for certain Nutritional Reference Values for subgroups of the population. Although FCN has not developed Nutritional Reference Values for all nutrients, this commission was included in our analysis, as the Nutritional Reference Values were established by experts from Switzerland and have been adopted by the Swiss authorities.

**Table 1:** Characteristics of the included societies included the type of documents provided to describe the DRV

Acronym of the societies	Full name of the societies and title of the recommendations	Countries	Languages of publications	Types of publications	Accessibility and price
ANSES	French Agency for Food, Environmental and Occupational Health & Safety	France	French	Different electronic reports	Free
SHC	Superior Health Council of Belgium Recommandations nutritionnelles pour la Belgique – 2016	Belgium	French	One unique electronic report for all nutrients	Free
D-A-CH	Nutrition Societies from Deutschland, Austria and Switzerland D-A-CH values	Germany, Austria and Switzerland	Mainly German or English	One main paper report and additional electronic reports for some nutrients and/or subgroups	Paper report with rational for DRV: 35 € Update for some nutrients: 5 € DRV (without rational) available online free of charge
EFSA	European Food Safety Authority Dietary reference values	European Union	English	One electronic report by nutrient	Free
FCN	Federal Commission for Nutrition	Switzerland	German or English or French	One electronic report by nutrient	Free
NNR	Nordic Co-operation 5^th^ Nordic Nutrition Recommendations	Denmark, Finland, Island, Norway, Sweden	English	One unique electronic report for all nutrients	Free
SACN	Scientific Advisory Committee on Nutrition	United Kingdom	English	One unique electronic report (COMA 1991) and updated reports for some nutrients (SACN)	Free
SINU	Società Italiana di Nutrizione Umana LARN – Livelli di assunzione di riferimento di nutrienti ed energia per la popolazione italiana	Italy	Italian	Book (672 pages), e-book	e-book: 29 € + tax book: 52 € + tax DRV (without rational) available online free of charge

COMA, Committee on Medical Aspects of Food and Nutrition Policy; DRV, Dietary Reference Values.

### Global analysis of the recommendations of scientific societies: Step 1

In addition to comparing the DRV of the included scientific societies, we analysed the general characteristics of the recommendations. The analysis focused on the different age groups and genders used by societies, accessibility and price of publications, language and type of publications, and the last updates of the recommendations.

### *Analysis of the* Dietary Reference Values *and methodologies: Step 1*

For the seven nutrients (i.e. protein, carbohydrates, folate, vitamin D, calcium, iodine and iron) analysed in Step 1, we extracted the DRV of the eight selected societies in an Excel file for the subgroups of the population, including infants, children, adolescents, adults and the elderly, by gender when appropriate. Inspired by the methodology used by Doets et al.^(19)^, in order to facilitate comparisons between societies, given that age ranges differed from one society to another, we selected specific age instead of using age ranges. We used five specific ages for the paediatric populations, namely 9 months, 2, 8, 11 and 16 years, four specific ages for adults, that is 20, 45, 55 and 65 years for the elderly.

For each nutrient and subgroup of the population, we calculated the median DRV (PRI when available or AI) of the eight societies and expressed the differences with the median in percentages. We considered a difference equal to or higher than ±20 % from the median as large, a difference between ±10 % and ±20 % as moderate, and a difference equal to or lower than ±10 % as small. For these seven nutrients, a figure showed the DRV of the eight societies by age. In addition, for each nutrient, we provided to the experts a table describing the types of methodologies and main sources used by each society to define the DRV for each subgroup.

### Getting the opinion of experts: Step 1

Based on a report containing analyses of the DRV of the seven nutrients, we developed an online survey to collect the opinions of experts. Five members of the research team pre-tested the questionnaires to check their structure, understanding of the questions and answer options and edited the English. The final questionnaire contained twelve questions addressing the (1) general presentation of the recommendations of the eight societies, (2) analysis of the methodologies used by the societies to define their DRV, (3) the extracted DRV, (4) general opinion and (5) general comments (Supplementary File I).

Then, the principal investigators of the project (SBDT and CJC) conducted individual interviews with each expert and discussed specific issues based on the findings of the survey. The interviews aimed to validate the preselection of the 2–4 societies and to reach a consensus on the methodology for Step 2. The interviews were recorded and lasted for approximately 1 h.

### *Analysis of the* Dietary Reference Values *and methodologies: Step 2*

In Step 2, the DRV from the two societies preselected during Step 1 were analysed. We extracted their DRV for forty-four macro- and micronutrients of the same subgroups of the population as in Step 1 and added the subgroups of pregnant women or breastfeeding women and the elderly aged 75 years specifically.

We calculated the differences between the DRV of the two societies. We considered a difference equal to or higher than ±15 % as large, a difference between ±10 % and ±15 % as moderate and a difference equal to or lower than ±10 % as small. We described the methodologies used by the two societies when a difference of ≥15 % in DRV was observed. We also compared the DRV and methodologies used to define the DRV of the two societies with those of the FCN.

### Getting the opinion of experts: Step 2

The experts received a report containing the analyses of the DRV of all nutrients analysed and completed a second online survey. The questions focused on (1) completeness of the reference values of the two preselected societies, (2) comparison of DRV and methodologies used by the two societies, (3) general opinions including the accessibility of basic data and (4) general comments (Supplementary File II). Five members of the research team pre-tested the questionnaire. After the survey, the principal investigators conducted a focus group with the six experts in order to discuss the final selection.

## Results

### Characteristics of the recommendations of the eight societies

The characteristics of the reference values of the eight societies are listed in Table 1. Most societies have published electronic scientific reports or publications containing the DRV and methodologies that are freely available online. The updates of the reference values differed. ANSES published new reference values containing DRV for micronutrients for all subgroups of the population in March 2021. NNR planned to update the DRV in 2022, and SACN updated the DRV for some nutrients, such as carbohydrates, vitamin D, folate, iodine and iron. The age ranges differed among societies, especially for paediatric subgroups and the elderly (Supplementary file III).

### *Analysis of* Dietary Reference Values *of protein and iron: Step 1*

As an illustration of the analysis of the DRV of the eight societies, we present the data for protein and iron. The same figures and tables were used for the other nutrients analysed in Step 1.

For proteins, most societies have expressed DRV in grams per kilogram of body weight (kg) per day. ANSES, EFSA, NNR and SHC recommended an amount of 0·83 g/kg per d for adults and the D-A-CH and FCN recommended 0·8 g/kg per d. SACN recommended the lowest value at 0·75 g/kg per d and SINU recommended the highest value at 0·9 g/kg per d. The DRV of all societies were higher for children and adolescents owing to growth. ANSES, D-A-CH, and FCN proposed higher DRV for the age group over 65 years.

**Fig. 2:**
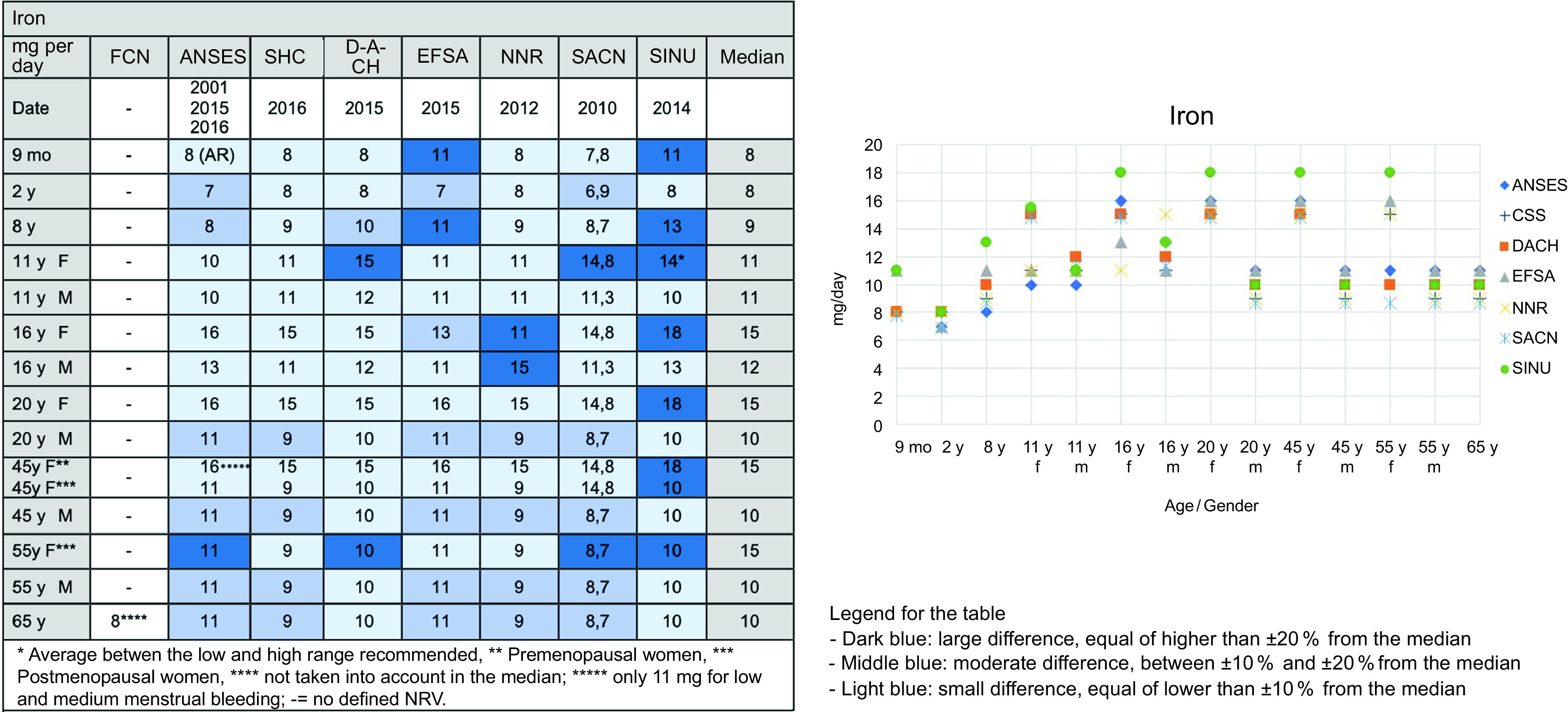
Example of the comparisons of DRV for iron from the eight preselected societies during Step 1 of the project performed from September 2020 to February 2021

For iron, as illustrated in Fig. 2, the DRV of all societies differed between women and men since adolescence until the age of menopause. For adult men, the DRV of all societies were quite similar. For women, large variations appeared between societies and depending on age groups. SINU recommended the highest values.

### Analysis of methodologies for protein and iron: Step 1

All societies have used various methodologies to define DRV depending on the nutrient and sometimes on the subgroups. To define the DRV for proteins, the societies used either their own methodology, and/or the one of EFSA, or WHO/FAO/UNU for some subgroups, as illustrated in Table 2. Most societies used their own methodologies for iron.

**Table 2:** General description of the type of methods used by the societies to define the DRV for protein and iron (Step 1)

Nutrient	Society	Date of publication	Method
Protein	ANSES	2016	Based on EFSA 2015
	SHC	2016	Based on EFSA 2012
	D-A-CH	2017	Own method and based on WHO/FAO/UNU 2007, for infants, children and adolescent
	EFSA	2012	Own method and based on WHO/FAO/UNU 2007, for infants, children and adolescent, and lactation
	FCN	2011	Based on WHO/FAO/UNU 2007
	NNR	2012	Own method and based on WHO/FAO/UNU 2007, for infants, children and adolescent, pregnancy and lactation
	COMA	1991	Based on WHO 1985
	SINU	2014	Own method and based scientific literature, WHO/FAO/UNU 2007 and EFSA 2012
Iron	ANSES	2016	Based on EFSA 2015
	SHC	2016	Own method
	D-A-CH	2015	Own method
	EFSA	2015	Own method
	FCN	–	–
	NNR	2012	Own method, based on NNR 2004
	SACN	2010	Own method, based on COMA 1991
	SINU	2014	Own method, based on scientific literature, WHO/FAO/UNU 2004 and Institute of Medicine 2001

EFSSA, European Food Safety Authority; COMA, Committee on Medical Aspects of Food and Nutrition Policy; DRV, dietary reference values; UNU, United Nations University.

### Experts’ opinion and preselection of societies: Step 1

In the first online survey and during the individual interviews, the experts agreed on several criteria for the preselection of societies for Step 2: (i) data and rationales for DRV were to be available in English or in all three national languages (i.e. French, German and Italian); (ii) published data had to be recent; (iii) with regular updates; (iv) and based on a solid and rigorous scientific methodology.

Based on these criteria and the analysis of the DRV and their methodology (contained in the intermediate report), all experts were clearly in favour of selecting EFSA and D-A-CH for Step 2. They recommended not selecting SACN/COMA, SINU, SHC or NNR because of problems with publication dates, language, accessibility or because their DRV were mainly based on another society. The experts favoured preselecting a third society, that is, ANSES, in case the EFSA and D-A-CH did not provide DRV for some nutrients, for some subgroups of the population, or had very different DRV.

After Step 1, the research team validated the decision to include EFSA, D-A-CH and ANSES in Step 2. However, at the beginning of Step 2, in March 2021, ANSES announced its decision to use the DRV of EFSA for all nutrients, except sodium. For this reason, we decided to perform the analyses of Step 2 only on the DRV of EFSA and D-A-CH.

Regarding the methodology planned for Step 2, the experts recommended analysing the completeness of the recommendations for the different nutrients and subgroups and comparing the values between the preselected societies. In the case of significant differences, they recommended searching for rationales used by each society.

### *Analysis of* Dietary Reference Values *of* European Food Safety Authority *and D-A-CH for all nutrients: Step 2*

The analysis of the completeness of the recommendations for the different nutrients and subgroups showed that EFSA and D-A-CH did not provide DRV for sugars, MUFA and cholesterol for all age categories (Table 3). D-A-CH did not provide DRV for carbohydrates and fibres for children and adolescents and eicosapentaenoic acid and DHA for all age categories. In addition, D-A-CH did not provide DRV for some nutrients for pregnant and breastfeeding women.

**Table 3:** Summary of the differences of DRV between EFSA and D-A-CH for macronutrients (step 2)

Macronutrients	Missing values: societies and subgroups	Difference ≥ 20 %	Difference between ≥ 10 and < 20 %
Carbohydrates	D-A-CH for children and adolescents		
Fibres	D-A-CH for children and adolescents	Adults; pregnancy; breastfeeding	
Sugars	Both for all categories		
MUFA	Both for categories		
Linoleic acid		All subgroups except 9 months	9 months
SFA	Both for all categories		
Eicosapentaenoic, docosahexaenoic acids	D-A-CH for all categories		
Cholesterol	Both for all categories		
Protein	D-A-CH for pregnancy: 1st trimester	65 y; 75 y	
Alcohol	EFSA for all categories		
Water		16 y (women); 55–75 y (men)	20 y (women); 2 y; 11 y (men); 20 y and 45 y (women)

DRV, Dietary Reference Values; Y, years.

The analysis of the comparison of DRV of EFSA and D-A-CH showed that only eight nutrients had a large difference of ≥20 % for all subgroups or the majority of subgroups: fibres, linoleic acid, pantothenic acid, vitamin D, chlorine, iodine, phosphorus and sodium (Tables 3 and 4). Other nutrients were different in some subgroups, mainly children, pregnant and breastfeeding women, and women or men. We did not observe any systematic differences between the DRV of EFSA and D-A-CH.

**Table 4:** Summary of the differences of DRV between EFSA and D-A-CH for micronutrients

Vitamins	Missing values: societies and subgroups	Difference ≥ 20 %	Difference between ≥ 10 and < 20 %
Biotin			16 y
*β*-Carotene	Both for all categories		
Cobalamin			Breastfeeding (2nd)
Folate			2 y; 8 y; 11 y; breastfeeding
Niacin	Both for all categories		
Pantothenic acid		8 y; 16 y; adults; pregnancy	Breastfeeding
Riboflavin	D-A-CH for pregnancy: 1st trimester	11 y; 16 y (women); adults (women); pregnancy; breastfeeding	2 y; 8 y (girls); adults (men)
Thiamine	D-A-CH for pregnancy: 1st trimester	9 mo; 2 y; 65 y (women); 75 y (women); pregnancy (2nd)	8 y; 20 y; 45 y; 55 y; 65 y (men); 75 y (men); pregnancy (2nd and 3nd); breastfeeding
Vitamin A		9 mo; 2 y; 16 y	8 y; 20 y, 45 y, & 55 y (men); pregnancy (2nd and 3nd)
Vitamin B6		16 y (women)	11 y; adults (women); pregnancy (1st)
Vitamin C		Breastfeeding	
Vitamin D		All subgroups except 9 mo	
Vitamin E		Breastfeeding	9 mo ; 2 y, 8 y, 16 y & 20 y (women); pregnancy
Vitamin K		2 y	11 y; 20 y, 45 y & 55 y (women); 65 y & 75 y (men); pregnancy; breastfeeding
Minerals and trace elements	Missing values: societies and subgroups	Difference ≥ 20 %	Difference between ≥ 10 and < 20 %
Calcium		2 y	9 mo; 8 y; pregnancy; breastfeeding
Chlorine		All subgroups	
Chromium	EFSA for all categories D-A-CH for pregnancy and breastfeeding		
Copper	D-A-CH for pregnancy and breastfeeding	9 mo 8 y, 45 y, 55 y & 65 y (men)	11y; 16y (women); pregnancy; breastfeeding
Fluoride		9 mo; 8 y	2 y; all adult (men)
Iodine		8 y; 11 y; 16 y; all adults; breastfeeding	9 mo; 2 y; pregnancy
Iron		9 mo; 11 y (girls)	2 y; 16 y (women)
Magnesium		breastfeeding	20 y (men)
Manganese	D-A-CH for pregnancy and breastfeeding	9 mo; 2 y; 8 y; 11 y	16 y; all adults; pregnancy; breastfeeding
Molybdenum	D-A-CH for pregnancy and breastfeeding	9 mo; 2 y; 8 y; 11 y	16 y; all adults; pregnancy; breastfeeding
Phosphorus		All subgroups	
Potassium		9 mo; 2 y	8 y; 16 y; all adults; pregnancy; breastfeeding
Selenium			8 y; 11 y; 16 y (women); adults (women); pregnancy; breastfeeding
Sodium		Children (except 9 mo); adults; pregnancy; breastfeeding	
Zinc		2 y; 11 y (girls); pregnancy (1st)	9 mo; 8 y; 11 y (boys); adults (women)

EFSSA, European Food Safety Authority; DRV, Dietary Reference Values; Mo, months; Y, years.

### *Analysis of methodologies used by* European Food Safety Authority *and D-A-CH for protein and iron: Step 2*

For pregnant and breastfeeding women, the D-A-CH^(20)^ recommended a total protein intake expressed in kg of body weight per day, while EFSA^(21)^ recommended the same values as for adults plus an additional amount in grams per day for the three trimesters of pregnancy. For the first trimester of pregnancy, D-A-CH considered that the small additional protein intake needed for pregnancy (0·4 g/d) could be neglected. For the age group over 65 years, EFSA considered that protein requirements in older adults were equal to those of adults based on nitrogen balance studies, that is 0·83 g/kg per d^(21)^. They used a similar approach to WHO/FAO/UNU^(22)^ which concluded that the available data did not provide convincing evidence that the protein requirement of elderly people differed from the protein requirement of younger adults. D-A-CH recommended higher DRV (1 g/kg per d) based on the results of studies showing better outcomes in the elderly with higher protein intake^(20)^. FCN used similar arguments to recommend an intake of 1·0–1·2 g/kg for healthy seniors and 1·2–1·5 for ageing people in frailty situation^(13)^.

For iron, both EFSA and D-A-CH determined the requirements using a factorial approach, based on the needs for growth, iron losses and bioavailability^(6,23)^. For adults, EFSA modelled whole-body iron losses using data from US adults. In premenopausal women, both EFSA and D-A-CH recommended higher intakes due to loss during menstruation (PRI at 16 and 15 mg/d, respectively). For pregnant and breastfeeding women, EFSA considered that iron stores and enhanced absorption provided sufficient additional iron and defined the same values as for premenopausal women^(23)^. D-A-CH recommended higher intakes than EFSA for pregnant and breastfeeding women, based on different sources including data from FAO/WHO 1988, US data and German studies^(6)^.

### Expert opinion and selection of societies: Step 2

In the second online survey focusing on the comparison between the D-A-CH and EFSA recommendations, the six experts rated the two societies quite similarly, with some nuances on specific topics. Considering completeness, four experts preferred EFSA for macronutrients and two had no preference. One expert preferred EFSA for vitamins, and five had no preference. None of the experts preferred a specific society for minerals and trace elements.

Based on the comparison of the DRV and the methodologies used, the experts had no preference for EFSA or D-A-CH for macronutrients (5/6 experts), minerals or trace elements (6/6 experts). Three experts preferred EFSA for vitamins, and three had no preference.

The accessibility of EFSA scientific reports and the completeness of EFSA recommendations were emphasised. The experts provided fifteen comments on energy and macronutrients, four comments on vitamins and seven comments on minerals and trace elements that were discussed during the focus group.

The focus group discussion included three main topics. Firstly, the experts debated the proposition of choosing EFSA as the main reference society for DRV. In their opinion, even if EFSA and D-A-CH would both qualify based on scientific evidence, the need for free and easily accessible scientific background information favoured EFSA. However, it also appeared that no society was optimal for all nutrients or subgroups of the population. Therefore, the second topic discussed by the experts concerned nutrients for which a recommendation other than EFSA would be required. In most of these cases, FCN had a recommendation, and D-A-CH was proposed for the remaining two cases. Finally, the third topic addressed during this focus group was the implementation of these new DRV. The experts shared global recommendations and highlighted the importance of communicating the new DRV to all stakeholders to ensure their adoption.

Based on the results of the 2-step methodology, the research group recommended FSVO selecting EFSA as the main reference society and using alternative societies (FCN and D-A-CH) for nine specific nutrients either for the overall population (for four nutrients) or subgroups (for three nutrients), and FCN for the elderly for all nutrients.

## Discussion

In this paper, we aimed to describe and discuss a 2-step methodology developed to select a reference society that provides DRV for national implementation and to illustrate its application to Switzerland with protein and iron. The 2-step methodology included an analysis of seven nutrients from eight societies and secondly an analysis of forty-four nutrients from two preselected societies. In each step, the research team analysed the DRV, the methodologies used to define the DRV, the practical aspects of the recommendations and the opinions of an external group of experts.

The 2-step methodology was a solution to overcome the challenges in selecting reference societies to provide DRV for Switzerland. The first issue was the large number of (1) nutrients including macro- and micronutrients, (2) population subgroups (infants, children, pregnant and breastfeeding women, etc.) and (3) DRV, such as PRI and AI. In addition, the definition of age groups differed among the scientific societies, as already observed by Doets et al.^(19)^ For instance, the age group of adolescents was defined for iron as 14–17 years by ANSES, NNR and SHC, as 15–17 years by SINU and as 12–17 years by EFSA. Finally, the DRV provided for a single nutrient occasionally differed among scientific societies. For example, D-A-CH, NNR and SACN provided a PRI for iodine, SHC, EFSA and SINU provided an AI, and ANSES provided both. This resulted in extensive and heterogeneous DRV to compare.

Adopting the 2-step methodology also simplified the process, as it would not have been manageable to simultaneously analyse the methodologies used by eight societies to define their DRV for forty-four nutrients and twelve population subgroups. The methodologies used to define DRV logically differ widely among nutrients. In addition, for some nutrients, the methodology varied depending on age group. As illustrated by the examples of protein and iron detailed in this paper, the methodology used was sometimes adopted from other societies, especially from EFSA and WHO/FAO, adapted from these societies to their own populations to a greater or lesser extent, or newly developed. For some nutrients or age groups, the descriptions of the methodologies used by each society were so intertwined that it was difficult to identify their exact sources or adaptations. It was also difficult to distinguish specific differences between societies.

In addition to the analyses of the values and methodologies conducted during the 2-step methodology, it was important to consider the general characteristics of DRV. This included, for example, the accessibility of scientific reports containing the methodologies used by each society to define their DRV^(24)^. For example, at the time of our project, the publications of D-A-CH were available free of charge and online only for certain nutrients, which influenced the final choice. Depending on the country, the existence of publications in each national language or in English is also essential to understand how DRV have been defined and ensure optimal implementation among professionals. This was particularly a key criterion for Switzerland, which has three linguistic regions. The dates of publication were also important criteria for selection, as well as the coming updates.

Developing new specific DRV from scratch requires time, technical and scientific competencies, leading to high costs and organisational complications^(25–27)^. These resources are necessary not only for the initial development but also for continuing updates. When choosing new DRV to be implemented at the national level, using existing ones from global scientific societies has several strategic advantages and is advocated by experts in the field^(25,27)^. For this reason, Switzerland has chosen this option. As a global society, EFSA accumulates many required assets, as highlighted by the findings of our 2-step methodology. In Europe, other countries, including France and Belgium, rely mainly on EFSA for their DRV. The trade-off of selecting a global society for national DRV inevitably includes the loss of national specificity, which may imply choosing alternative societies for some key nutrients or subgroups of the population. As an example, France chose an alternative recommendation for sodium in their 2021 publication instead of EFSA for the rest of the micronutrients. Similarly, in Switzerland, we also chose alternative societies for some nutrients, such as sugar, MUFA and SFA and alcohol, for which EFSA did not provide DRV. We also selected FCN for the subgroup of the elderly, based on a published report which provided specific DRV for vulnerable and non-vulnerable populations^(13)^.

Allen et al.^(26)^ already mentioned that adapting current recommendations from different sources is a pragmatic approach considering that physiological requirements vary little across populations globally, and setting reference values requires determining an acceptable level of uncertainty. This group of authors have published a useful tool kit including a flow diagram, which guides in the process of deciding which approach is best, i.e. adapting existing DRV or establishing new ones^(27)^.

This study has some limitations. First, we used the median DRV of the eight societies for each nutrient and subgroup of the population to express the differences between societies during Step 1. Although the median is less influenced by extreme values than the mean, it may not capture the complete heterogeneity of the DRV of different societies. However, this method provides a clear summary of the similarities and discrepancies among the DRV. The second limitation is linked to the methodologies used by scientific societies to define DRV. They frequently adopted DRV from WHO/FAO/UNU (worldwide) or Institute of Medicine (US), whereas we voluntarily included European societies providing DRV for a population similar to the Swiss population. Despite these limitations, this project used a structured approach, based on objective criteria, and included an external group of national experts to select societies in two steps. Consensus among the research group and external experts was reached quite rapidly. The implication of different key stakeholders in this process is important for optimising the future implementation of these new DRV at the national level^(28)^.

In conclusion, to deal with the large heterogeneity and complexity of data, it was very helpful to adopt a 2-step methodology including fewer nutrients and more societies during Step 1 and fewer societies but all nutrients in Step 2. We selected EFSA as the main society to provide DRV for Switzerland and alternative societies for specific nutrients and subgroups of the population. This transparent 2-step methodology may be applied by other countries that consider an update and/or national harmonisation of their DRV using existing ones from reference scientific societies.

## Supporting information

Jotterand Chaparro et al. supplementary material Jotterand Chaparro et al. supplementary material

## Supplementary material

For supplementary material accompanying this paper visit https://doi.org/10.1017/S1368980023002902.
