# Supplementary material for: Development and application of a 2-step methodology to select a reference society providing Dietary Reference Values for national implementation
Source: Public Health Nutr. 2024 Jan 2;27(1):e28. doi: 10.1017/S1368980023002902 (PMC10830378; doi:10.1017/S1368980023002902)
Supplement: Jotterand Chaparro et al. supplementary material [file S1368980023002902sup001.pdf]

Supplementary files

Supplementary file I: Questionnaire used to conduct the online survey during Step 1

**General presentation of the recommendations of societies**	
1. To what extent do you think that the publications related to DRVs from the following society are sufficiently up-to-date to be included?	
- ANSES	☐ Yes, absolutely ☐ Rather yes ☐ Rather no ☐Not at all
- SHC	☐ Yes, absolutely ☐ Rather yes ☐ Rather no ☐Not at all
- D-A-CH	☐ Yes, absolutely ☐ Rather yes ☐ Rather no ☐Not at all
- EFSA	☐ Yes, absolutely ☐ Rather yes ☐ Rather no ☐Not at all
- NNR	☐ Yes, absolutely ☐ Rather yes ☐ Rather no ☐Not at all
- SACN/COMA	☐ Yes, absolutely ☐ Rather yes ☐ Rather no ☐Not at all
- SINU	☐ Yes, absolutely ☐ Rather yes ☐ Rather no ☐Not at all
2. To what extent do you think a society that publishes only in one (or mostly in one) of the national languages can be selected?	☐ Yes, absolutely ☐ Rather yes ☐ Rather no ☐Not at all
3. To what extent do you think a society that publishes only in English can be selected?	☐ Yes, absolutely ☐ Rather yes ☐ Rather no ☐Not at all
**Analysis of the methodologies used by the societies to define their DRVs**	
4. Based on the methodologies used by each of the societies below to define the DRVs for the 7 nutrients, to what extent would you select this society?	
- ANSES	☐ Yes, absolutely ☐ Rather yes ☐ Rather no ☐Not at all
- SHC	☐ Yes, absolutely ☐ Rather yes ☐ Rather no ☐Not at all
- D-A-CH	☐ Yes, absolutely ☐ Rather yes ☐ Rather no ☐Not at all
- EFSA	☐ Yes, absolutely ☐ Rather yes ☐ Rather no ☐Not at all
- NNR	☐ Yes, absolutely ☐ Rather yes ☐ Rather no ☐Not at all
- SACN/COMA	☐ Yes, absolutely ☐ Rather yes ☐ Rather no ☐Not at all
- SINU	☐ Yes, absolutely ☐ Rather yes ☐ Rather no ☐Not at all
5. To what extent do you think that a society that uses predominantly the methodologies and DRVs of another society can be selected?	☐ Yes, absolutely ☐ Rather yes ☐ Rather no ☐Not at all
6. Is there one or more subgroup(s) of the population for which the methodology seems inadequate?<br>If yes, for which subgroup(s), nutrient (s) and for which society-ies?	☐ No ☐ Yes
7. Is there one or more nutrient(s) for which the methodology seems inadequate?<br>If yes, for which one(s) and for which society-ies?	☐ No ☐ Yes

**Analysis of the DRVs**	
8. Following the analysis of the DRVs defined for the 7 nutrients (values themselves), do you think that the following society could provide suitable DRVs for Switzerland?<br>- ANSES<br>- SHC<br>- D-A-CH<br>- EFSA<br>- NNR<br>- SACN/COMA<br>- SINU	 <br><br><br><br>☐ Yes, absolutely ☐ Rather yes ☐ Rather no ☐Not at all<br>☐ Yes, absolutely ☐ Rather yes ☐ Rather no ☐Not at all<br>☐ Yes, absolutely ☐ Rather yes ☐ Rather no ☐Not at all<br>☐ Yes, absolutely ☐ Rather yes ☐ Rather no ☐Not at all<br>☐ Yes, absolutely ☐ Rather yes ☐ Rather no ☐Not at all<br>☐ Yes, absolutely ☐ Rather yes ☐ Rather no ☐Not at all<br>☐ Yes, absolutely ☐ Rather yes ☐ Rather no ☐Not at all
9. Do you have any comments about the DRV of one or more nutrients?	Free answer
**General opinion**	
10. Based on the intermediate report, to what extent do you think the following societies should be preselected for Step 2 analysis?<br>- ANSES<br>- SHC<br>- D-A-CH<br>- EFSA<br>- NNR<br>- SACN/COMA<br>- SINU	 <br><br><br>☐ Rather yes ☐ Rather no ☐ I can't say<br>☐ Rather yes ☐ Rather no ☐ I can't say<br>☐ Rather yes ☐ Rather no ☐ I can't say<br>☐ Rather yes ☐ Rather no ☐ I can't say<br>☐ Rather yes ☐ Rather no ☐ I can't say<br>☐ Rather yes ☐ Rather no ☐ I can't say<br>☐ Rather yes ☐ Rather no ☐ I can't say
11. Based on the intermediate report, what would be your order of preference for the preselection of the societies for Step 2 analysis?	Arrange the seven societies according to your order of preference (1 = the most appropriate; 7 = the least appropriate)
**General comments**	
12. Do you have any general comments on the project (analysis, methodology, next step…) that may be discussed during the individual interviews?	Free answer

Supplementary file II: Questionnaire used to conduct the online survey during Step 2

**Completeness of the recommendations of the two societies**	
1. Based on the completeness of the recommendations of the two societies for **macronutrients**, do you think the following society may be selected?<br>- D-A-CH<br>- EFSA	 <br><br>☐ Yes, absolutely ☐ Rather yes ☐ Rather no ☐Not at all<br>☐ Yes, absolutely ☐ Rather yes ☐ Rather no ☐Not at all
2. Based on the completeness of the recommendations of the two societies for **macronutrients**, which society would you prefer?	☐ D-A-CH ☐ EFSA ☐ Both
3. Based on the completeness of the recommendations of the two societies for **macronutrients**, do you think some nutrients may be problematic?	☐ Yes ☐ No
4. If yes, which **macronutrients**? And what would you propose?	Free answer
5. Based on the completeness of the recommendations of the two societies for **vitamins**, do you think the following society may be selected?<br>- D-A-CH<br>- EFSA	 <br><br>☐ Yes, absolutely ☐ Rather yes ☐ Rather no ☐Not at all<br>☐ Yes, absolutely ☐ Rather yes ☐ Rather no ☐Not at all
6. Based on the completeness of the recommendations of the two societies for **vitamins**, which society would you prefer?	☐ D-A-CH ☐ EFSA ☐ Both
7. Based on the completeness of the recommendations of the two societies for **vitamins**, do you think some nutrients may be problematic?	☐ Yes ☐ No
8. If yes, which **vitamins**? And what would you propose?	Free answer
9. Based on the completeness of the recommendations of the two societies for **minerals and trace elements**, do you think the following society may be selected?<br>- D-A-CH<br>- EFSA	 <br><br>☐ Yes, absolutely ☐ Rather yes ☐ Rather no ☐Not at all<br>☐ Yes, absolutely ☐ Rather yes ☐ Rather no ☐Not at all
10. Based on the completeness of the recommendations of the two societies for **minerals and trace elements**, which society would you prefer?	☐ D-A-CH ☐ EFSA ☐ Both
11. Based on the completeness of the recommendations of the two societies for **minerals and trace elements**, do you think some nutrients may be problematic?	☐ Yes ☐ No

12. If yes, which **minerals and trace elements**?<br>And what would you propose?	Free answer
**Comparison of DRVs and methodologies used by the societies**	
13. Based on the comparison of DRVs and methodologies used by the two societies for **macronutrients**, do you think the following society may be selected?<br>- D-A-CH<br>- EFSA	 <br>☐ Yes, absolutely ☐ Rather yes ☐ Rather no ☐Not at all<br>☐ Yes, absolutely ☐ Rather yes ☐ Rather no ☐Not at all
14. Based on the comparison of DRVs and methodologies used by two societies for **macronutrients**, which society would you prefer?	☐ D-A-CH ☐ EFSA ☐ Both
15. Based on the comparison of DRVs and methodologies used by the two societies for **macronutrients**, do you think some nutrients may be problematic?	☐ Yes ☐ No
16.If yes, which **macronutrients**?<br>And what would you propose?	Free answer
17. Based on the comparison of DRVs and methodologies used by the two societies for **vitamins**, do you think the following society may be selected?<br>- D-A-CH<br>- EFSA	 <br>☐ Yes, absolutely ☐ Rather yes ☐ Rather no ☐Not at all<br>☐ Yes, absolutely ☐ Rather yes ☐ Rather no ☐Not at all
18. Based on the comparison of DRVs and methodologies used by two societies for **vitamins**, which society would you prefer?	☐ D-A-CH ☐ EFSA ☐ Both
19. Based on the comparison of DRVs and methodologies used by the two societies for **vitamins**, do you think some nutrients may be problematic?	☐ Yes ☐ No
20. If yes, which **vitamins**?<br>And what would you propose?	Free answer
21. Based on the comparison of DRVs and methodologies used by the two societies for **minerals and trace elements**, do you think the following society may be selected?<br>- D-A-CH<br>- EFSA	 <br>☐ Yes, absolutely ☐ Rather yes ☐ Rather no ☐Not at all<br>☐ Yes, absolutely ☐ Rather yes ☐ Rather no ☐Not at all
22. Based on the comparison of DRVs and methodologies used by two societies for **minerals and trace elements**, which society would you prefer?	☐ D-A-CH ☐ EFSA ☐ Both

23. Based on the comparison of DRVs and methodologies used by the two societies for **minerals and trace elements**, do you think some nutrients may be problematic?	☐ Yes ☐ No
24. If yes, which **minerals and trace elements**?<br>And what would you propose?	Free answer
25. For all nutrients, we have considered a difference between the DRVs of the two societies ≥15% as scientifically significant. For you, what would be a clinically significantly cut-off?	Free answer
**General opinion including accessibility of basic data**	
26. Which society would you select considering the following criteria:<br>- Accessibility of scientific reports<br>- Completeness of recommendations<br>- Comparison of DRVs and methodologies used by the two societies<br>- Credibility of the society<br>- Acceptability of the new DRVs in comparison with the current values of the FCN<br>- Applicability in practice of the DRVs in Switzerland including consistency with legislation	☐ D-A-CH ☐ EFSA ☐ Both<br>☐ D-A-CH ☐ EFSA ☐ Both<br>☐ D-A-CH ☐ EFSA ☐ Both<br>☐ D-A-CH ☐ EFSA ☐ Both<br>☐ D-A-CH ☐ EFSA ☐ Both<br>☐ D-A-CH ☐ EFSA ☐ Both
27. Overall, based on the 2nd intermediate report, which society would you prefer?	☐ D-A-CH ☐ EFSA ☐ Both
28. Do you have any general comments about the choice of the society?	Free answer
**General comments**	
29. Do you have any general comments on the project that should be discussed during the focus group?	Free answer

Supplementary file III: Definitions of population subgroups by the different societies

**Societies**	**Age group: 0 – 12 months**	**Age group: 1 – 18 years**	**Age group: adults (years)**	**Upper age group (years)**	**Other groups**
**ANSES**	0-5, 6-11	12-35, 4-5, 6-9, 10-13, 14-17	> 18	> 65	Pregnancy Breastfeeding
**SHC** Example for calcium	0-5, 6-11	1-3, 4-6, 7-10, 11-14, 15-18	18-60	> 60	Pregnancy Breastfeeding
Example for iron	0-3, 5-6, 7-12	1-3, 4-5, 6-9, 10-13, 14-17	18-30, 31-60, 61-71	> 74	Pregnancy Breastfeeding
**D-A-CH**	0–3, 4–12	1–3, 4–6, 7–9, 10–12, 13–14, 15–18	19–24, 25–50, 51–64	≥ 65	
**EFSA** Example for calcium	7-11	1-3, 4-10, 11-17	18-24, > 25		
Example for protein	6-12	18 months, 2, 3 → 17	18-59	> 60	Pregnancy: trimester 1 to 3 Breastfeeding: semester 1 to 2
**FNC** Example for fluor	0-4, 4-12	1-4, 4-7, 7-10, 10-13, 13-15, 15-19	19-25, 25-51, 51-65	> 65	Pregnancy Breastfeeding
**NNR** Macronutrients	6-12	12-23 months			Pregnancy Breastfeeding
Micronutrients	< 6, 6-12	12-23 months, 2-5, 6-9, 10-13, 14-17	18-30, 31-60, 61-74	> 74	
**SACN**		1, 2-3, 4-6, 7-10, 11-14, 15-18	19-64, 65-74	> 75	
**SINU**	6-12	1-3, 4-6, 7-10, 11-14, 15-17	18-19, 30-59, 60-74	> 75	Pregnancy: trimester 1 to 3 Breastfeeding: semester 1 to 2
